# Mental health at work: WHO guidelines

**DOI:** 10.1002/wps.21094

**Published:** 2023-05-09

**Authors:** Aiysha Malik, José Luis Ayuso‐Mateos, Gergő Baranyi, Corrado Barbui, Graham Thornicroft, Mark van Ommeren, Aemal Akhtar

**Affiliations:** ^1^ Department of Mental Health and Substance Use World Health Organization Geneva Switzerland; ^2^ Centro de Investigación Biomédica en Red de Salud Mental Instituto de Salud Carlos III Madrid Spain; ^3^ Department of Psychiatry Universidad Autónoma de Madrid Madrid Spain; ^4^ Centre for Research on Environment, Society and Health, Department of Geography University of Edinburgh Edinburgh UK; ^5^ WHO Collaborating Centre for Research and Training in Mental Health and Service Evaluation; Department of Neuroscience, Biomedicine and Movement Sciences, Section of Psychiatry University of Verona Verona Italy; ^6^ Centre for Global Mental Health and Centre for Implementation Science Institute of Psychiatry, Psychology and Neuroscience, King's College London London UK; ^7^ Department of Clinical Neuroscience, Division of Insurance Medicine Karolinska Institutet Stockholm Sweden; ^8^ School of Psychology, University of New South Wales Sydney Australia

Globally, 60% of people work^1^, and an estimated 15% of working‐age adults have a mental disorder at any point in time^2^, with a likely higher rate in people with an increased exposure to risk factors for mental health at work, such as those facing inadequate pay or job insecurity. People living with severe mental health conditions face exclusion from work, largely due to stigma and discrimination, although participation in work activities is important for recovery^3^. Poor mental health can diminish a person's identity at work, reduce productivity and increase absenteeism, with depression and anxiety alone estimated to determine 12 billion lost workdays per year, impacting the global economy annually by nearly 1 trillion USD^2^.

The right to good health, including mental health, and the right to decent work are fundamental human rights. Policies which support workers’ well‐being are essential to advance progress towards the United Nations Sustainable Development Goals 3 and 8. Despite international conventions calling for protection of physical and mental health^4^, focus within occupational health has largely been on physical health, and few countries have work‐related mental health prevention and promotion programmes^5^. In response to this burden and limited action, the World Health Organization (WHO) has developed guidelines^6^ that provide recommendations to effectively address mental health at work.

The guidelines have been developed through methods outlined in the WHO handbook for guideline development^7^. WHO guidelines utilize PICO (Population/Problem ‐ Intervention ‐ Comparison ‐ Outcome) questions, identified in collaboration with topic experts who form the Guideline Development Group. Systematic reviews of best available evidence are conducted, prioritizing randomized controlled studies where feasible, addressing critical outcomes. GRADE (Grading of Recommendations, Assessment, Development, and Evaluations) methodology evaluates the certainty of available evidence. Recommendations balance benefits against harms, and consider beneficiaries’ values, implementation feasibility, resources required, cost‐effectiveness, health equity, equality and discrimination, human rights and socio‐cultural aspects.

The WHO Guidelines on Mental Health at Work address organizational interventions, manager and worker training, individual interventions, return to work, recovery‐oriented strategies, and screening programmes. Recommendations are provided for universal interventions; interventions for health, humanitarian and emergency workers; and interventions for workers with mental health conditions. Based on this, thirteen evidence profiles have been developed^6^.

To prevent risks to mental health at work, the WHO guidelines recommend organizational interventions – approaches targeting the mitigation, reduction or removal of psychosocial risk factors (e.g., bullying, low job control). Organizational interventions help reduce emotional distress and improve work‐related outcomes, including absenteeism, job satisfaction and work performance. These interventions are best delivered through meaningful participation of workers. However, most of the reviewed research evidence in this area has been found to be of very low quality, likely due to challenges of evaluating these highly complex interventions. Methodological rigor must be prioritized to bolster this evidence base, which exemplifies an opportunity to target determinants of mental health.

To protect and promote mental health at work, the WHO recommends the provision of mental health training to managers, aimed to strengthen their mental health‐related knowledge, attitudes and skills, and improve workers’ help‐seeking. Such training equips managers to identify and support workers who experience distress, and address stressors related to working conditions. These trainings are not intended for managers to become mental health care providers. No recommendations have been made regarding leadership‐oriented training, as evidence did not document clear effects on health outcomes. Training for workers largely targets mental health literacy and awareness. This was found to reduce stigmatizing attitudes and improve mental health‐related knowledge, but, though such training is popular, there was no substantiated effect on mental health symptoms or help‐seeking.

The guidelines also recommend individual interventions, such as psychosocial interventions or physical activity, which promote positive mental health, reduce levels of emotional distress, and improve work‐related outcomes such as work‐effectiveness. Workers’ values demonstrated that they perceive individual interventions as an indication that they are singularly responsible for their mental health. Consequently, these interventions should be made available as one component of a comprehensive programme which includes organizational and managerial approaches.

To support people with mental health conditions to participate in work, reasonable accommodations which adapt working environments to match capacities and preferences of workers are recommended, in line with promotion of human rights^8^. The Guideline Development Group considered return‐to‐work programmes following an absence associated with mental health conditions. Evidence‐based mental health clinical care, in combination with work‐directed care (e.g., graded return to work) or alone, leads to reductions in mental health symptoms and absence. Recovery‐oriented strategies focusing on vocational and economic inclusion, such as (augmented) supported employment, are effective for persons with severe mental health conditions in obtaining and maintaining employment.

No recommendation was made for screening programmes during employment (screening plus follow‐up support), owing to the uncertainty on whether the benefits outweigh the harms. The statement of no recommendation does not apply to screening as required by some occupational regulations.

The WHO Guidelines on Mental Health at Work are based on the best available recent evidence, yet a substantial research‐gaps agenda is proposed to address the limited high‐quality and un‐diverse research. Work‐related outcomes are often absent from research on mental health at work. Science must go beyond defining psychosocial risks at work, to develop high‐quality evidence on what organizational approaches work for whom. The world's largest working populations remain unusually under‐researched, including informal workers, and those who work in small‐ and medium‐sized enterprises and in low‐ and middle‐income countries.

A WHO and International Labour Organization joint policy brief was released alongside the guidelines to support stakeholders in their application^9^. This brief provides a roadmap to improve mental health at work through creating an enabling environment for prevention of exposure to risks, protection and promotion of mental health at work, and support for people with mental health conditions to participate and thrive at work.
